# Genome-wide CRISPR screens identify PTPN21 and WDR26 as modulators of the mitochondrial stress-induced ISR

**DOI:** 10.1093/lifemeta/loae020

**Published:** 2024-05-28

**Authors:** Wen Li, Mingyue Dong, Kaiyu Gao, Jialiang Guan, Ying Liu

**Affiliations:** State Key Laboratory of Membrane Biology, New Cornerstone Science Laboratory, Institute of Molecular Medicine, College of Future Technology, Peking University, Beijing 100871, China; Peking-Tsinghua Center for Life Sciences, Academy for Advanced Interdisciplinary Studies, Peking University, Beijing 100871, China; State Key Laboratory of Membrane Biology, New Cornerstone Science Laboratory, Institute of Molecular Medicine, College of Future Technology, Peking University, Beijing 100871, China; Peking-Tsinghua Center for Life Sciences, Academy for Advanced Interdisciplinary Studies, Peking University, Beijing 100871, China; State Key Laboratory of Membrane Biology, New Cornerstone Science Laboratory, Institute of Molecular Medicine, College of Future Technology, Peking University, Beijing 100871, China; State Key Laboratory of Membrane Biology, New Cornerstone Science Laboratory, Institute of Molecular Medicine, College of Future Technology, Peking University, Beijing 100871, China; PKU-Tsinghua-NIBS Graduate Program, Academy for Advanced Interdisciplinary Studies, Peking University, Beijing 100871, China; State Key Laboratory of Membrane Biology, New Cornerstone Science Laboratory, Institute of Molecular Medicine, College of Future Technology, Peking University, Beijing 100871, China; Peking-Tsinghua Center for Life Sciences, Academy for Advanced Interdisciplinary Studies, Peking University, Beijing 100871, China; Beijing Advanced Innovation Center for Genomics, Beijing 100871, China

**Dear Editor,**

Organisms have evolved mitochondrial stress response pathways to surveil mitochondrial function and activate repair programs upon detection of mitochondrial dysfunction [1]. Failure to respond to mitochondrial perturbation has been implicated in aging and age-related diseases [2, 3]. Mitochondrial perturbation in mammals primarily activates the integrated stress response (ISR), in which four kinases—HRI (haem-regulated inhibitor or eukaryotic translation initiation factor 2 alpha (eIF2α) kinase 1 (EIF2AK1)), PERK (PKR-like ER kinase), GCN2 (general control nonderepressible 2), and PKR (protein kinase RNA-dependent)—mediate the phosphorylation of eIF2α [4–6]. eIF2α phosphorylation selectively elevates the translation of a subset of transcripts, including those encoding the transcription factors ATF4 (activating transcription factor 4) and CHOP (C/EBP homologous protein) [7, 8]. Since the gene that encodes CHOP is also a target of ATF4 [9], transcription of *CHOP* is also upregulated. Here, we employed genome-wide CRISPR/Cas9 screens to identify potential regulators of the ISR during mitochondrial stress in mammalian cells. We then focused on tyrosine-protein phosphatase nonreceptor type 21 (*PTPN21*) and WD repeat-containing protein 26 (*WDR26*), two of the candidate genes from the screen. Knockdown of *PTPN21* or *WDR26* suppressed the induction of ISR upon mitochondrial perturbation. Mechanistically, PTPN21 and WDR26 facilitated the interaction between DELE1 (death-associated protein 3 (DAP3)-binding cell death enhancer 1) and HRI, thereby relaying the mitochondrial stress signal to the cytosol. Deficiency of *PTPN21* or *WDR26* impaired cell fitness upon challenge with mitochondrial stressors. These findings pave the way to understanding the mechanisms underlying the mitochondrial stress-induced ISR in mammalian cells.

To identify potential factors that mediate the mitochondrial stress-induced ISR in mammalian cells, we sought to perform an unbiased genetic screen with the use of CRISPR/Cas9 technology. As previously reported, treating 293T cells with different mitochondrial inhibitors—paraquat, antimycin, or carbonyl cyanide *m*-chlorophenyl hydrazone (CCCP)—induced the expression of the transcription factor CHOP [10, 11] (Fig. 1a–c; Supplementary Fig. S1a). We then performed immunostaining using a commercial antibody against CHOP and found that the fluorescence signal was greatly induced when cells were treated with the mitochondrial stressor antimycin (Supplementary Fig. S1b). Fluorescence-activated cell sorting (FACS) successfully separated untreated or antimycin-treated cells based on CHOP fluorescence intensity (Supplementary Fig. S1c). Most importantly, when *CHOP* knockout cells were treated with antimycin, they showed same FACS profile as untreated cells (Fig. 1d). We then performed FACS-based genome-wide CRISPR screens in the presence of antimycin to identify positive and negative regulators of the mammalian mitochondrial stress response (Fig. 1e). The top and bottom 5% of the cells were sorted out based on CHOP fluorescence intensity. Genomic DNA was extracted, PCR-amplified, and subjected to sequencing for single guide RNA (sgRNA) enrichment analysis (Fig. 1e). Two datasets were generated based on our analysis, showing the normalized abundance of each sgRNA in two biological replicates within the “Ctrl” and “CHOP-low” or “CHOP-high” groups (Supplementary Tables S1 and S2). Candidate genes were picked based on the following criteria: the normalized sgRNA count in the “Ctrl” dataset was not < 7, and the sgRNA was enriched over 2-fold in both biological replicates. Notably, while we were working on this project, two elegant studies were published using similar CRISPR approaches to monitor the induction of ATF4 or CHOP and search for regulators of ISR [12, 13]. Both studies identified a previously less-characterized protein, DELE1, which transduces mitochondrial stress signals to the cytosol to activate HRI kinase and initiate ISR. Encouragingly, in our screen, DELE1 and HRI were also identified to be required for the induction of CHOP (Fig. 1f; Supplementary Table S1). These results indicate that our CRISPR screens successfully uncover regulators of the mitochondrial stress-induced ISR in mammalian cells.

**Figure 1:**
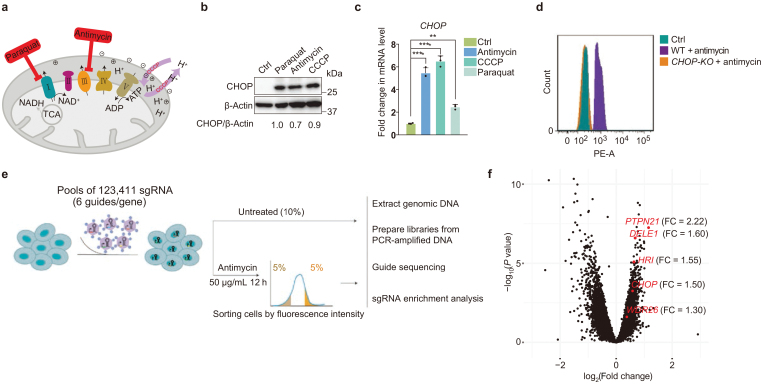
Genome-wide CRISPR screens to identify modulators of the mitochondrial stress response. (a) Schematic depiction of different mitochondrial inhibitors. Paraquat inhibits the function of mitochondrial complex I, antimycin is an inhibitor of mitochondrial complex III, and CCCP is an uncoupler of mitochondrial oxidative phosphorylation. (b) Immunoblotting analysis showing that different mitochondrial inhibitors induce the expression of CHOP. Paraquat, 0.5 mg/mL 12 h; antimycin, 50 μg/mL 12 h; CCCP, 10 μmol/L 12 h. (c) qRT-PCR measures *CHOP* mRNA levels in HEK293T cells treated with different mitochondrial inhibitors. Quantifications are normalized to *ACTIN*. Data are presented as mean ± SEM. *n* = 3 biological replicates. ^**^P < 0.01, ^****^P < 0.0001 by one-way ANOVA test. (d) Flow cytometry reveals that antimycin treatment induces the upregulation of CHOP. Wild-type HEK293T cells and *CHOP* knockout (KO) cells serve as control groups. (e) Schematic diagram of the procedure for the genome-wide CRISPR screens. (f) Genome-wide screens uncover regulators of the mitochondrial stress response in mammalian cells. The positive fold changes represent genes enriched in the “CHOP-low” group.

Among the candidate genes, we first focused on *PTPN21* because the fold enrichment of this gene was high and knockdown of *PTPN21* by small interfering RNA (siRNA) or short hairpin RNA (shRNA) indeed impaired the induction of CHOP at both the protein and mRNA levels when cells were challenged with antimycin (Fig. 2a and b; Supplementary Fig. S2a and b). In addition, knockdown of *PTPN21* resulted in suppression of CHOP induction when cells were treated with other mitochondrial inhibitors such as CCCP and oligomycin (Supplementary Fig. S2c–f). Furthermore, deficiency of *PTPN21* inhibited the induction of ATF4, another transcription factor involved in the ISR (Supplementary Fig. S2g). Expression of PTPN21 in *PTPN21*-knockdown cells restored the induction of CHOP (Fig. 2c), excluding the possibility that the small RNAs generate off-target effects. Importantly, deficiency of *PTPN21* not only disrupted the induction of CHOP and ATF4, but also impaired the upregulation of multiple genes that are involved in the mitochondrial stress response (Fig. 2d; Supplementary Fig. S2h) [14, 15].

**Figure 2:**
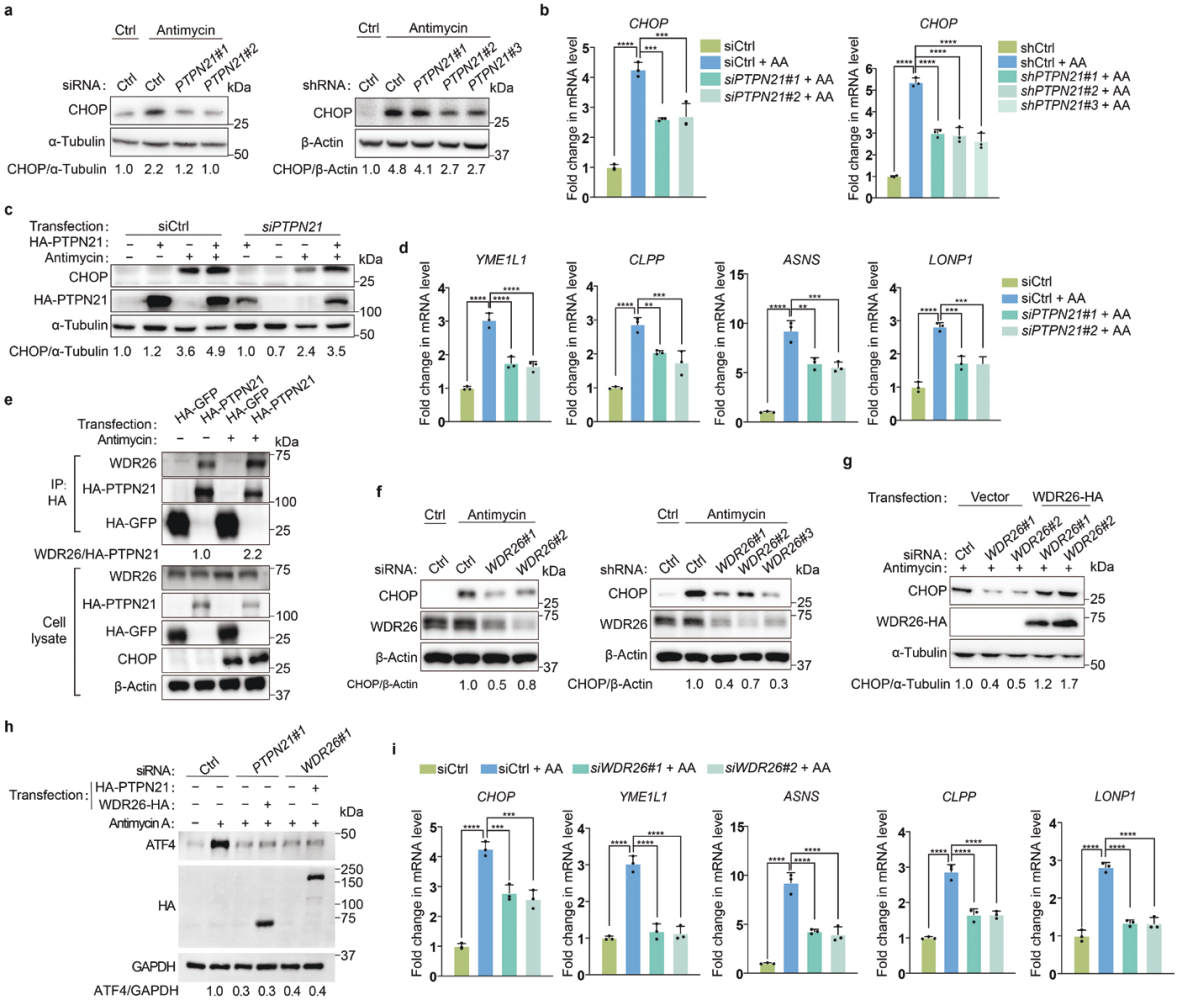
PTPN21 and WDR26 regulate the mitochondrial stress response. (a) Knockdown of *PTPN21* by siRNA or shRNA suppresses the induction of CHOP upon mitochondrial stress. (b) qRT-PCR measures *CHOP* mRNA levels in wild-type cells or *PTPN21* knockdown cells under normal conditions or mitochondrial stress conditions. (c) Overexpression of *PTPN21* restores the induction of CHOP in *PTPN21*-deficient cells under mitochondrial stress. (d) qRT-PCR measures mRNA levels of mitochondrial stress response genes under the indicated conditions. (e) Immunoprecipitation experiments reveal that the interaction between PTPN21 and WDR26 is enhanced upon mitochondrial dysfunction. (f) Knockdown of *WDR26* by siRNA or shRNA suppresses the induction of CHOP upon mitochondrial stress. (g) Overexpression of *WDR26* restores the induction of CHOP in *WDR26*-deficient cells under mitochondrial stress. (h) Knockdown of *PTPN21* or *WDR26* blocked the upregulation of the ISR induced by overexpression of *WDR26* or *PTPN21*. (i) qRT-PCR measures mRNA levels of mitochondrial stress response genes under the indicated conditions. Quantifications are normalized to *ACTIN*. Data are presented as mean ± SEM. *n* = 3 biological replicates. ^**^P < 0.01, ^***^P < 0.001, ^****^P < 0.0001 by one-way ANOVA test. Cells were treated with 50 μg/mL of antimycin for 12 h.

To further explore the function of PTPN21 in mitochondrial stress-induced ISR, we searched the BioGRID database to see whether any PTPN21-associated proteins were also identified in our CRISPR screen. WDR26, a protein that interacts with PTPN21, was uncovered by our screen. Interestingly, the interaction between WDR26 and PTPN21 was enhanced upon mitochondrial perturbation (Fig. 2e). Knockdown of *WDR26* by siRNA or shRNA also inhibited the induction of CHOP and ATF4 under mitochondrial stress conditions (Fig. 2f; Supplementary Fig. S2c–g). Overexpression of *WDR26* dramatically enhanced the expression of CHOP in *WDR26*-deficient cells (Fig. 2g). More importantly, the upregulation of the ISR induced by overexpression of *PTPN21* or *WDR26* was completely blocked by knockdown of the corresponding other gene (*WDR26* or *PTPN21*), suggesting that these two proteins collaboratively modulate the ISR in response to mitochondrial stress (Fig. 2h). Additionally, knockdown of *WDR26* impaired the induction of other mitochondrial stress response genes (Fig. 2i; Supplementary Fig. S2h and i). Conversely, knockdown of *PTPN21* or *WDR26* did not impair the induction of the endoplasmic reticulum (ER) stress marker gene *BIP* (binding immunoglobulin protein) (Supplementary Fig. S2j). Moreover, knockdown of *PTPN21* or *WDR26* did not inhibit the activation of the ISR induced by nutrient deprivation (Supplementary Fig. S2k). Taken together, these results indicate that PTPN21 and WDR26 play important roles in the mitochondrial stress-induced ISR in mammalian cells.

In mammalian cells, mitochondrial dysfunction promotes the cleavage of DELE1 protein by mitochondrial protease OMA1 (overlapping with the *m*-AAA protease 1 homolog), resulting in accumulation of cleaved DELE1 in the cytosol, where it associates with HRI and stimulates the kinase activity of HRI to phosphorylate eIF2α (Fig. 3a) [12, 13]. Enhanced eIF2α phosphorylation then facilitates the translation and expression of CHOP [8]. In line with these discoveries, treating cells with an ISR inhibitor (ISRIB), which reverses the effects of eIF2α phosphorylation, or overexpression of *GADD34* (growth arrest and DNA damage-inducible protein 34, a phosphatase of eIF2α), resulted in the suppression of CHOP induction upon antimycin treatment (Fig. 3b; Supplementary Fig. S3a). In addition, the elevated induction of CHOP in *PTPN21*-overexpressing cells treated with antimycin was compromised by ISRIB or *GADD34* overexpression (Fig. 3b; Supplementary Fig. S3a). More importantly, knockdown of *PTPN21* or *WDR26* suppressed the induction of eIF2α phosphorylation and enhanced global protein synthesis upon mitochondrial inhibition (Fig. 3c and d). These data suggest that PTPN21 and WDR26 function upstream of eIF2α phosphorylation during the mitochondrial stress-induced ISR.

**Figure 3:**
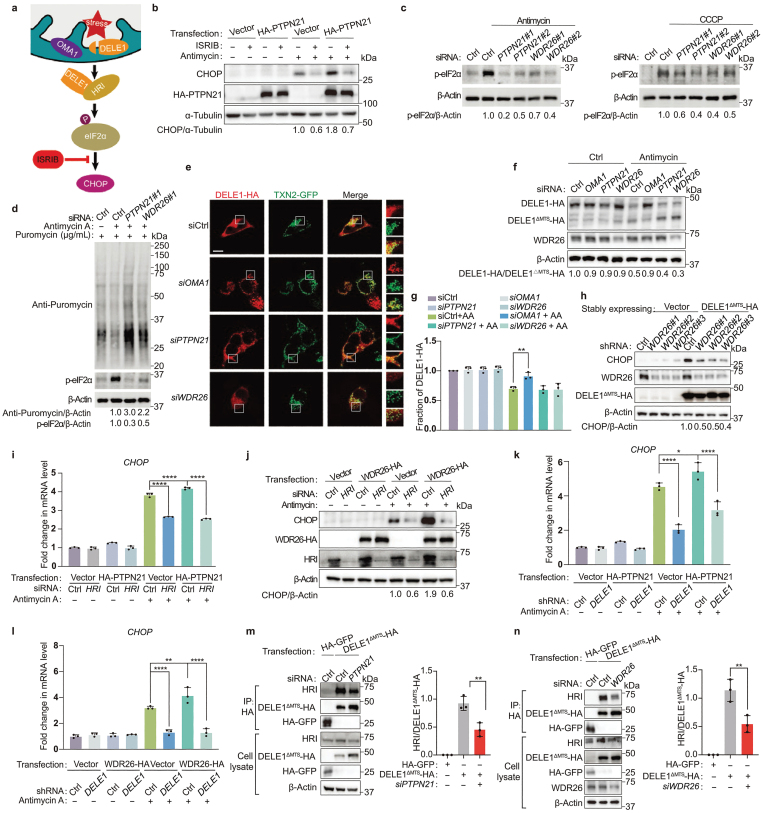
PTPN21 and WDR26 promote the interaction between DELE1 and HRI. (a) Model of the mitochondrial stress response pathway. (b) ISRIB suppresses the induction of CHOP in cells overexpressing *PTPN21*. (c) Knockdown of *PTPN21* or *WDR26* suppresses the induction of eIF2α phosphorylation in response to mitochondrial stress. Antimycin, 50 μg/mL 4 h; CCCP, 10 μmol/L 4 h. (d) Knockdown of *PTPN21* or *WDR26* enhances global protein synthesis. (e) Representative fluorescence images of DELE1 localization. Before capturing the images, HeLa cells were treated with 50 μg/mL of antimycin for a duration of 3 h. Scale bar, 20 µm. (f) Immunoblotting analysis reveals that the cleavage of DELE1 is not affected by knocking down *PTPN21* or *WDR26*. (g) Quantifications of DELE1 cleavage in f. *n* = 3 biological replications. ^**^P < 0.01 by one-way ANOVA test. (h) WDR26 acts downstream of cleaved DELE1. (i and j) Knockdown of *HRI* suppresses the induction of CHOP upregulation in cells with overexpression of *PTPN21* (i) or *WDR26* (j). *n* = 3 biological replications. ^****^P < 0.0001 by one-way ANOVA test. (k and l) The induction of *CHOP* expression in cells with *PTPN21* (k) or *WDR26* (l) overexpression is suppressed by knockdown of *DELE1*. *n* = 3 biological replications. ^*^P < 0.05, ^**^P < 0.01, ^****^P < 0.0001 by one-way ANOVA test. (m and n) Knockdown of *PTPN21* (m) or *WDR26* (n) impairs the interaction between HRI and DELE1 lacking the mitochondrial targeting sequence (DELE1^ΔMTS^). The relative levels of HRI/DELE1^ΔMTS^-HA were measured and quantified across three independent replicates. *n* = 3 biological replications. Data are presented as mean ± SEM. ^**^P < 0.01 by two-sided Student’s *t*-test.

Consistent with previously published results [12, 13], when cells were treated with the mitochondrial inhibitors antimycin or CCCP, DELE1 started to accumulate in the cytosol (Supplementary Fig. S3b and c), and cytosolic accumulation of DELE1 was blocked by knockdown of the mitochondrial protease *OMA1* (Fig. 3e). Knockdown of *PTPN21* or *WDR26* did not alter the mitochondrial localization of DELE1 under normal conditions (Supplementary Fig. S3d), or the cytosolic accumulation of DELE1 under mitochondrial stress conditions (Fig. 3e). In addition, unlike *OMA1*, knockdown of *PTPN21* or *WDR26* did not prevent the cleavage of DELE1 (Fig. 3f and g). Furthermore, expression of DELE1 lacking the mitochondrial targeting sequence (*DELE1*^*ΔMTS*^) dramatically elevated the expression of CHOP, even in the absence of mitochondrial inhibitors (Fig. 3h; Supplementary Fig. S3e). Knockdown of *WDR26* or *PTPN21* suppressed the induction of CHOP in cells overexpressing *DELE1*^*ΔMTS*^ (Fig. 3h; Supplementary Fig. S3e). These results indicate that PTPN21 and WDR26 function after the cleaved DELE1 accumulates in the cytosol. Consistent with these findings, we observed that PTPN21 and WDR26 constantly localized in the cytosol, regardless of the presence or absence of mitochondrial stress inducers (Supplementary Fig. S3f).

To further dissect the site of action of PTPN21 and WDR26, we performed epistatic analysis to see whether they functioned upstream or downstream of HRI. Overexpression of *PTPN21* or *WDR26* elevated CHOP expression in antimycin-treated cells (Fig. 3i and j). However, the upregulated expression of CHOP in cells overexpressing *PTPN21* or *WDR26* was compromised by knockdown of *HRI* or *DELE1* (Fig. 3i–l). Once cleaved DELE1 accumulated in the cytosol, it interacted with HRI to transduce the mitochondrial stress signal (Supplementary Fig. S3g). Based on the above findings, we then tested whether PTPN21 and WDR26 affected the interaction between DELE1 and HRI. Knockdown of *PTPN21* or *WDR26* indeed suppressed the interaction between DELE1 and HRI (Fig. 3m and n). Taken together, these results indicate that PTPN21 and WDR26 facilitate the interaction between HRI and cleaved DELE1, thereby activating HRI to phosphorylate eIF2α and promote CHOP expression (Supplementary Fig. S3h).

Consistent with the important roles of PTPN21 and WDR26 in mediating the mitochondrial stress-induced ISR, knockdown of *PTPN21* or *WDR26* greatly altered mitochondrial morphology when cells were challenged with the mitochondrial stressor antimycin. Upon antimycin treatment, *PTPN21* or *WDR26* knockdown cells exhibited more extensive mitochondrial morphology disruption compared to wild-type cells. This suggests a reduced resilience and capacity in these cells to manage mitochondrial damage (Fig. 4a and b). Knockdown of *HRI*, *PTPN21*, or *WDR26* also modestly affected mitochondrial respiration, as evidenced by the reductions in basal respiration, maximal respiration, and ATP production (Supplementary Fig. S4a and b). These results suggest that these proteins play a fundamental role in maintaining mitochondrial functionality in the cells.

**Figure 4:**
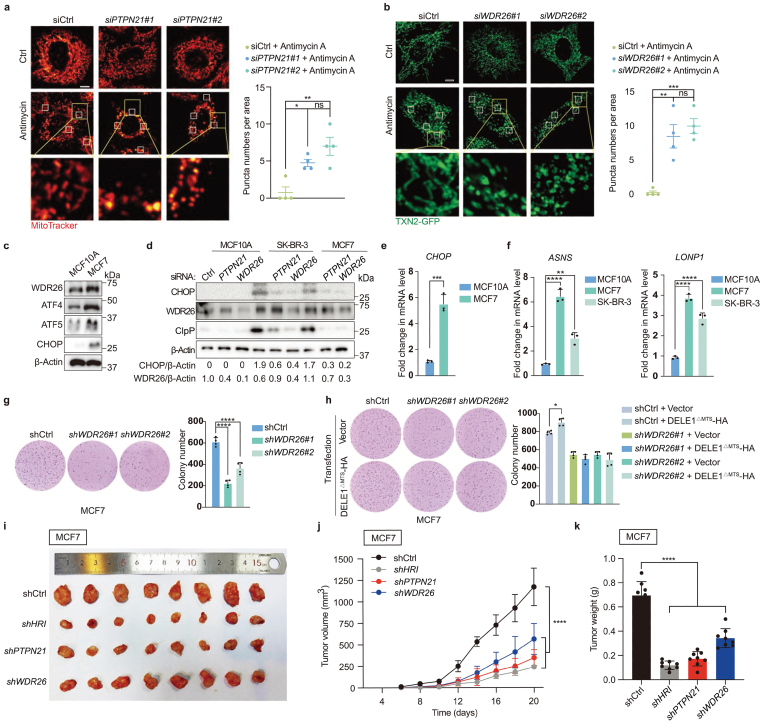
PTPN21 and WDR26 regulate mitochondrial physiology and cell fitness. (a and b) Representative fluorescence images to reveal mitochondrial morphology in HeLa cells stained with MitoTracker or transfected with TXN2 (Thioredoxin 2)-GFP. Cells were transfected with *PTPN21* siRNA (a) or *WDR26* siRNA (b) and cultured in the presence or absence of antimycin. Scale bar, 10 µm. Quantification of punctate mitochondria was conducted in four smaller squares of the same area in the specified Hela cells. ^*^P < 0.05, ^**^P < 0.01, ^***^P < 0.001 by one-way ANOVA test. (c) The expression levels of WDR26, ATF4, ATF5, and CHOP in MCF10A and MCF7 cells. (d) Immunoblotting experiments reveal the expression levels of CHOP, ClpP, and WDR26 in breast cancer cells. (e) qRT-PCR measures the mRNA levels of *CHOP* in MCF10A and MCF7 cells. Quantifications are normalized to *ACTIN*. Data are presented as mean ± SEM. *n* = 3 biological replicates. ^***^P < 0.001 by two-sided Student’s *t*-test. (f) qRT-PCR measures mRNA levels of *ASNA* and *LONP1* in breast cancer cells. Quantifications are normalized to *ACTIN*. Data are presented as mean ± SEM. *n* = 3 biological replicates. ^**^P < 0.01, ^****^P < 0.0001 by one-way ANOVA test. (g) Deficiency of *WDR26* suppresses anchorage-independent cell growth in soft agar. Colony numbers were counted and quantified. *n* = 4 biological replications. ^****^P < 0.0001 by one-way ANOVA test. (h) Overexpression of cleaved DELE1 promotes anchorage-independent cell growth in wild-type cells, but not in *WDR26*-deficient cells. *n* = 4 biological replicates. ^*^P < 0.05 by one-way ANOVA test. (i−k) Deficiency of *PTPN21* or *WDR26* inhibits subcutaneous xenograft tumor growth of MCF7 cells. MCF7 cells stably infected with shCtrl, *shHRI*, *shPTPN21*, or *shWDR26* were subcutaneously injected into nude mice for xenograft growth. The tumor images (i), the growth curve of tumor volume (j), and the tumor weight (k) are shown. Tumor volume and tumor weight are quantified (*n* = 8). Data are mean ± SEM. ^****^P < 0.0001 by one-way ANOVA test.

Upregulation of WDR26 has been linked with the onset of breast cancer and poor survival rate of breast cancer patients [16]. Through bioinformatic analysis, we also noticed that the *WDR26* gene had the highest copy number in breast cancer samples (Supplementary Fig. S4c). Analysis of the Gene Expression Profiling Interactive Analysis (GEPIA) 2 database also revealed higher expression of the *WDR26* gene in breast cancer tumors, compared with normal tissues (Supplementary Fig. S4d). Consistent with the above analysis, the expression levels of WDR26 protein in the breast cancer cell lines MCF7 and SK-BR-3 were much higher than that of the breast epithelial cell line MCF10A (Fig. 4c and d). Moreover, the transcript and protein levels of CHOP and other mitochondrial stress response genes were also higher in breast cancer cells (Fig. 4d–f). Additionally, knockdown of *WDR26* in MCF7 and SK-BR-3 cells suppressed the elevated expression of CHOP and ClpP (caseinolytic protease proteolytic subunit) (Fig. 4d).

Anchorage-independent growth, a key trait of metastatic potential, reflects the ability of cells to grow without attachment, influenced by cellular stress responses. Mitochondrial stress responses, often induced in tumor environments by conditions like nutrient deprivation or hypoxia, lead to essential cellular adaptations and metabolic reprogramming for cancer cell survival and proliferation [17]. Given this context, we explored whether WDR26, important in mitochondrial stress-induced ISR, impacted the anchorage-independent growth of cancer cells. Our findings revealed that *WDR26* knockdown in MCF7 and SK-BR-3 cells significantly inhibited their anchorage-independent growth (Fig. 4g). More importantly, although expression of DELE1^ΔMTS^ significantly promoted anchorage-independent cell growth of wild-type MCF7 and SK-BR-3 cells, it did not enhance the growth of *WDR26*-deficient MCF7 and SK-BR-3 cells (Fig. 4h; Supplementary Fig. S4e). Accordingly, knockdown of *PTPN21* also prevented anchorage-independent cell growth of MCF7 and SK-BR-3 cells (Supplementary Fig. S4e–g), and overexpression of *DELE1*^*ΔMTS*^ did not promote the growth of *PTPN21*-deficient MCF7 and SK-BR-3 cells (Supplementary Fig. S4e and g). Consistent with the above findings, knockdown of *HRI*, a known component of the mitochondrial stress-induced ISR in mammals, similarly hindered anchorage-independent cell growth of *DELE1*^*ΔMTS*^-overexpressing MCF7 and SK-BR-3 cells (Supplementary Fig. S4h and i). To further evaluate the role of PTPN21 or WDR26 in cancer development, we used a xenograft model to assess the *in vivo* effects of inhibiting these genes. The results showed that xenograft tumors in the *shPTPN21*, *shWDR26*, and *shHRI* groups were significantly smaller in both volume and weight compared to the control group (Fig. 4i–k). Taken together, these results suggest that WDR26 and PTPN21 modulate cell fitness and tumor growth through regulation of the mitochondrial stress-induced ISR.

Mitochondrial malfunction has been linked with numerous diseases including neurodegenerative disorders. Cells activate the mitochondrial stress response pathways to cope with the ever-changing environment and alleviate mitochondrial stress. To achieve unbiased identification of genes involved in the mitochondrial stress-induced ISR in mammals, we performed genome-wide CRISPR/Cas9 screens to uncover genes required for the induction of CHOP expression in the presence of a mitochondrial inhibitor. We then focused on PTPN21 and WDR26, and demonstrated that these two proteins promote the interaction between DELE1 and HRI to activate the ISR. Lastly, we showed that PTPN21 and WDR26 play crucial roles in regulating mitochondrial physiology and cell fitness.

Mitochondria play a crucial role in producing ATP through oxidative phosphorylation and synthesizing essential cellular components including amino acids, fatty acids, cholesterol, heme, nucleotides, and intermediate metabolites that can function as signaling molecules. As a result, cancer cells heavily rely on mitochondrial functions to maintain their elevated levels of proliferation, metastasis, and drug resistance [18]. The mitochondrial stress-induced ISR ensures the maintenance of ATP production and the biosynthesis of macromolecules even under unfavorable conditions, providing cancer cells with the energy and nutrients needed for their uncontrolled growth. Targeting mitochondrial metabolism has emerged as a potential strategy for cancer therapy [19]. PTPN21 is a cytosolic nonreceptor tyrosine phosphatase isolated from human skeletal muscle. The precise role of PTPN21 is still not fully understood, but recent reports suggest that it plays an important role in cell adhesion, growth, scattering, and migration [20–22]. WDR26 belongs to the family of WD-40 repeat proteins, which are highly conserved across various eukaryotic species [23]. The involvement of WDR26 in oxidative stress-induced cell injury and intellectual disabilities has been suggested [24–27], although its exact function remains unclear. Our studies indicate critical roles for PTPN21 and WDR26 in the mitochondrial stress-induced ISR in mammals. Moreover, we demonstrate that knockdown of *PTPN21* or *WDR26* effectively inhibits the growth of breast cancer cells.

The mitochondrial stress response has been extensively studied in the model organism *Caenorhabditis elegans*. A mitochondrion-to-nucleus communication pathway, named the mitochondrial unfolded protein response (UPR^mt^), is initiated upon mitochondrial perturbation. Through the use of fluorescent reporter strains, in which the expression of green fluorescent protein (GFP) is driven by the promoter of the mitochondrion-specific chaperone gene *hsp-6* (heat shock protein 6) or *hsp-60*, multiple genes were identified to be required for UPR^mt^ induction [28–34]. Among these regulators, ATFS-1 (activating transcription factor associated with stress-1) holds particular importance due to its ability to function as a transcription factor. Notably, upon mitochondrial dysfunction, ATFS-1 can accumulate in the cytosol and subsequently translocate into the nucleus, enabling it to transmit stress signals and reprogram the transcriptiome [12, 13, 35]. ATF5 (activating transcription factor 5) has been suggested as an ortholog of ATFS-1 in mammalian cells [36]. However, unlike ATFS-1, ATF5 lacks direct capability to relay mitochondrial stress signals to the nucleus. In mammals, DELE1 undergoes cleavage and subsequent transportation to the cytosol, where it interacts with the kinase HRI, resulting in the activation of ISR and the induction of several transcription factors, such as CHOP, ATF4, and ATF5 [12, 13]. The mechanism by which DELE1 exquisitely transduces the stress signal requires further exploration.

In summary, we identified PTPN21 and WDR26 as regulators of ISR during mitochondrial stress. Knockdown of these genes suppresses ISR induction by disrupting the interaction between DELE1 and HRI. Deficiency of *PTPN21* or *WDR26* impairs cell fitness under mitochondrial stress. The identification of a group of genes by our screen will pave the way for a detailed understanding of the mitochondrial stress-induced ISR in mammals. The gene lists will also offer potential targets for the treatment of neurodegenerative diseases and age-related disorders.

## Supplementary Material

loae020_suppl_Supplementary_Materials

loae020_suppl_Supplementary_Table_S1

loae020_suppl_Supplementary_Table_S2

## Data Availability

The authors declare that all data supporting the findings of this study are available within the paper and/or the supplementary information.

## Code availability

No custom code was generated or applied in this study.
