# Supplementary material for: Genome-wide CRISPR screens identify PTPN21 and WDR26 as modulators of the mitochondrial stress-induced ISR
Source: Life Metab. 2024 May 28;3(4):loae020. doi: 10.1093/lifemeta/loae020 (PMC11749115; doi:10.1093/lifemeta/loae020)
Supplement: loae020_suppl_Supplementary_Materials [file loae020_suppl_Supplementary_Materials.docx]

Supplementary Materials for

**Genome-wide CRISPR screens identify PTPN21 and WDR26 as modulators of the mitochondrial stress-induced ISR**

Materials and methods

Antibodies

Anti-CHOP (CST L63F7; 1:1,000 dilution in immunoblotting), anti-CHOP (Abcam ab11419; 1:100 dilution in immunostaining), anti-β-Actin (CST 13E5, 1:10,000 dilution in immunoblotting), anti-HRI (Santa Cruz sc-365239; 1:1,000 dilution in immunoblotting), anti-HA (CST C29F4; 1:3000 dilution in immunoblotting, 1:100 dilution in immunostaining), anti-WDR26 (ABclonal A10594; 1:1,000 dilution in immunoblotting), anti-α-Tubulin (CST 2144; 1:1,000 dilution in immunoblotting), anti-GFP (Abcam ab290; 1:1,000 dilution in immunostaining), anti-ATF4 (Beyotime AF2560; 1:1,000 dilution in immunoblotting), anti-ATF5 (Beyotime AF2563; 1:1,000 dilution in immunoblotting), anti-CLPP (Proteintech 15698-1-AP; 1:1,000 dilution in immunoblotting), anti-Phospho-eIF2α (Ser51) (CST 3398S; 1:1,000 dilution in immunoblotting), anti-Phospho-p70 S6 Kinase (Thr389) (CST 9234S; 1:1,000 dilution in immunoblotting), anti-GAPDH (Proteintech CL594-60004; 1:5,000 dilution in immunoblotting), anti-Puromycin (ABclonal A21205; 1:2,000 dilution in immunoblotting), anti-Myc (ImmunoWay YM3203, 1:2,500 dilution in immunoblotting), and anti-HSP60 (CST 4870S; 1:1,000 dilution in immunoblotting). Secondary antibodies (sigma, A4416-1ML; Jackson ImmunoResearch, 111-035-003; ABclonal, HRP Goat Anti-Rabbit IgG (H+L) (AS014) at 1:10000 dilution in immunoblotting).

Cell lines and cell culture

MCF10A, MCF7, and SK-BR-3 cells were provided by Prof. Gaoliang Ouyang (Xiamen University). HEK293T cells and HeLa cells were obtained from the American Type Culture Collection (ATCC, Manassas, VA, USA). MCF10A cells were cultured in mammary epithelial cell basal medium (MEBM) medium (Lonza) supplemented with its additives (without using GA-1000) and 100 ng/mL cholera toxin (Sigma). MCF7 cells were cultured in Eagle's minimum essential medium (EMEM) (ATCC), supplemented with 10% fetal bovine serum (FBS), penicillin, and streptomycin (P/S). HEK293T cells, HeLa cells, and SK-BR-3 cells were cultured in Dulbecco's modified Eagle medium (DMEM) high glucose medium (Gibco), supplemented with 10% FBS (Gibco) and penicillin/streptomycin (HyClone). All cell lines were maintained at 37°C and 5% CO_2_. For drug treatments, antimycin (AA, Sigma A8674) was dissolved in ethanol, and CCCP (Sigma C2759) and paraquat (Sigma 856177) were dissolved in water. Oligomycin (MedChemExpress, HY-N6782), Tunicamycin (Beyotime, SC0393), and ISRIB (Selleck Chemicals, S7400) were dissolved in DMSO. Puromycin Dihydrochloride (Beyotime, ST551-10mg) was used directly. All drugs were diluted to the indicated concentrations in the culture medium.

Expression of cDNA, siRNA, or shRNA in cells

Stable cell lines were generated by lentivirus infection. Three lentiviral expression constructs, pBOBI-DELE1-HA, pBOBI-DELE1^△MTS^-HA, and pBOBI-TXN2-GFP, were used. To generate pBOBI-DELE1-HA and pBOBI-TXN2-GFP, *DELE1* and *TXN2* cDNAs were PCR-amplified from total RNA isolated from HEK293T cells, and then ligated into the pBOBI vector containing a CMV promoter. pBOBI-DELE1^△MTS^-HA was then created from pBOBI-DELE1-HA by deleting the first 115 codons of *DELE1*. A total of 1 × 10^6^ HEK293T cells in a 6-well plate were transfected with a plasmid mixture containing pMDLg/pRRE, pRSV-Rev, pCI-VSVG, and target plasmid. Eight hours after transfection, the cell culture medium was replaced with a fresh medium. Cells were cultured for another 24 h to ensure virus production. The supernatant was then collected. A total of 5 × 10^5^ HEK293T cells in a 12-well plate were cultured with the virus-containing medium supplemented with 8 μg/mL polybrene for 24 h. G418 was added to the medium for selection.

For shRNA expression in cells, packaging of the virus was carried out as described above. The cells were infected with the virus. 2 μg/mL puromycin was added to the culture medium for positive selection. Sequences of shRNAs were as follows:

shWDR26#1:

5’CCGGCCTCAGATGATGGCACTGTTACTCGAGTAACAGTGCCATCATCTGAGGTTTTTTG3’

5’AATTCAAAAACCTCAGATGATGGCACTGTTACTCGAGTAACAGTGCCATCATCTGAGG3’;

shWDR26#2:

5’CCGGGCAGTTCTATCAGTGTGACTTCTCGAGAAGTCACACTGATAGAACTGCTTTTTTG3’

5’AATTCAAAAAGCAGTTCTATCAGTGTGACTTCTCGAGAAGTCACACTGATAGAACTGC3’;

shWDR26#3:

5’CCGGCCAGATGACAACTATCTTGTTCTCGAGAACAAGATAGTTGTCATCTGGTTTTTTG3’

5’AATTCAAAAACCAGATGACAACTATCTTGTTCTCGAGAACAAGATAGTTGTCATCTGG3’;

shPTPN21#1:

5’CCGGCAGCACCAACTCCTTAAATAACTCGAGTTATTTAAGGAGTTGGTGCTGTTTTT3’

5’AATTCAAAAACAGCACCAACTCCTTAAATAACTCGAGTTATTTAAGGAGTTGGTGCTG3’;

shPTPN21#2:

5’CCGGGACGCCATACAAATAGCACAACTCGAGTTGTGCTATTTGTATGGCGTCTTTTT3’

5’AATTCAAAAAGACGCCATACAAATAGCACAACTCGAGTTGTGCTATTTGTATGGCGTC3’;

shPTPN21#3:

5’CCGGCAACGAATGATGAAAGGTGTACTCGAGTACACCTTTCATCATTCGTTGTTTTT3’

5’AATTCAAAAACAACGAATGATGAAAGGTGTACTCGAGTACACCTTTCATCATTCGTTG3’

shDELE1#1:

5’ CCGGCCTTTGTCTTTGAGCAAATAACTCGAGTTATTTGCTCAAAGACAAAGGTTTTTTG3’

5’AATTCAAAAAACCTTTGTCTTTGAGCAAATAACTCGAGTTATTTGCTCAAAGACAAAGG3’;

shDELE1#2:

5’ CCGGGAGTGTTGTAAGACTAGGTTTCTCGAGAAACCTAGTCTTACAACACTCTTTTTTG3’

5’AATTCAAAAAAGAGTGTTGTAAGACTAGGTTTCTCGAGAAACCTAGTCTTACAACACT3’;

shHRI#1:

5’ CCGGGTACAATGCTTCGTTGTATTTCTCGAGAAATACAACGAAGCATTGTACTTTTTTG3’

5’AATTCAAAAAAGTACAATGCTTCGTTGTATTTCTCGAGAAATACAACGAAGCATTGTAC3’;

shHRI#2:

5’ CCGGGCAGAAGTTCTAACAGGTTTACTCGAGTAAACCTGTTAGAACTTCTGCTTTTTTG3’

5’AATTCAAAAAAGCAGAAGTTCTAACAGGTTTACTCGAGTAAACCTGTTAGAACTTCTGC3’;

shHRI#3:

5’ CCGGCAGAGAGCAATGTGGTGTTAACTCGAGTTAACACCACATTGCTCTCTGTTTTTTG3’

5’AATTCAAAAAACAGAGAGCAATGTGGTGTTAACTCGAGTTAACACCACATTGCTCTCTG3’.

For siRNA-mediated knockdown, siRNAs were transfected using the RNAiMAX transfection reagent (Thermo). Sequences of siRNAs were as follows:

siCtrl: 5’CGUACGCGGAAUACUUCGA3’

*siPTPN21#1*: 5’GCAUAGAUUUCUAUCUUAATT3’

*siPTPN21#2*: 5’GGAGACCAUUCAAUUUCAATT3’

*siWDR26#1*: 5’CAAACGUUGUUGGGAAUAATT3’

*siWDR26#2*: 5’GCAGGCGGUGGAACUACAATT3’

*siHRI#1*: 5’GGCGUAAUUCCCACCUAGATT3’

*siHRI#2*: 5’GAUUAAGGGUGCAACUAAATT3’

*siOMA1#1*: 5’GAUCAAUUGGGUUAUUCAUTT3’

*siOMA1#2*: 5’CAAGUUAACCAUAUAGUAATT3’

CRISPR/Cas9 screen

The genome-scale CRISPR/Cas9 knockout library (GeCKO v2, Addgene #1000000048) constructed using the backbone plasmid lentiCRISPR v2 (Addgene #52961) was used. HEK293T cells were transfected with the human GeCKO v2 library at a viral titer of 0.3, then cultured in a medium containing puromycin to select successfully transfected cells. For screening, the puromycin-resistant cells were treated with 50 μg/mL antimycin for 12 h, harvested and fixed with 4% paraformaldehyde, and then stained with anti-CHOP antibodies. Antimycin-treated cells were fluorescence-activated cell sorting (FACS)-sorted based on the ratio of fluorescence intensity, and the populations corresponding to the top 5% and bottom 5% of cells were collected. After that, genomic DNA was extracted. The inserted sgRNA fragment was amplified and sequenced with next-generation sequencing. Two datasets were generated based on our analysis, showing the normalized abundance of each sgRNA in two biological replicates within the “Ctrl” and “CHOP-high” or “CHOP-low” groups. Candidate genes were picked based on the following criteria: 1) the normalized sgRNA count in the “Ctrl” group was not less than 7, and 2) the sgRNA was enriched over 2-fold in both biological replicates.

Immunoblotting

HEK293T cells were lysed on ice for 10 min with RIPA lysis buffer (20 mmol/L Tris-HCl, 150 mmol/L NaCl, 1 mmol/L Na_2_EDTA, 1 mmol/L EGTA, 1% NP-40, 1% sodium deoxycholate, 2.5 mmol/L sodium pyrophosphate, 1 mmol/L β-glycerophosphate, 1% SDS supplemented with EDTA-free protease inhibitors). Cell lysates were centrifuged at 12,000 rpm for 10 min at 4°C. Supernatants were dissolved in Laemmli Sample Buffer (Bio-Rad), and then boiled at 95°C for 10 min. Samples were separated by SDS-PAGE and transferred onto a PVDF membrane (Bio-Rad). The membrane was blocked with 5% milk, incubated overnight at 4°C with primary antibodies, and incubated with secondary antibodies for 2 h at room temperature. Membranes were developed with the enhanced chemiluminescence method (Thermo) and visualized using the Tanon 5200 chemical luminescence imaging system or visualized by Kodak films.

Immunoprecipitation

Cells were lysed in TX-100 lysis buffer (20 mmol/L Tris-HCl, 150 mmol/L NaCl, 1 mmol/L Na_2_EDTA, 1 mmol/L EGTA, 2.5 mmol/L sodium pyrophosphate, 1 mmol/L β-glycerophosphate, 1% Triton X-100, protease inhibitors, pH 7.4), and incubated on ice for 30 min. Cell lysates were clarified by centrifuging at 12,000 rpm for 10 min at 4 °C. Totally, 90 μL aliquot of the clarified supernatant was prepared as the whole cell lysate sample. The remaining lysate was combined with washed anti-HA resin (Sigma) for immunoprecipitation, rotated at 4°C for 2 h, and washed three times with TX-100 lysis buffer. Finally, the resin-immobilized proteins were denatured and dissolved in Laemmli Sample Buffer (Bio-Rad) by boiling at 95°C for 10 min.

For the overexpression-based experiments, HEK293T cells were transfected with pcDNA3.3-based expression vectors using a polyethyleneimine (PEI) transfection reagent. Eight hours after transfection, the culture medium was replaced with a fresh medium. Cells were cultured for another 36 h to allow protein expression. Cells were then lysed and processed as described above.

Immunostaining

Cells were plated on polylysine-coated glass coverslips (Corning) and cultured overnight. After drug treatment, cells were washed with PBS, fixed with 4% paraformaldehyde in PBS for 10 min, washed gently with PBS, permeabilized with 0.1% Triton X-100 in PBS for 10 min, washed again with PBS, and blocked with 5% BSA for 30 min at room temperature. The cells were then incubated overnight at 4°C with primary antibodies (1:200 dilutions), washed gently with PBS, incubated with secondary antibodies in PBS (1:200 dilutions) for 2 h at room temperature, and washed again with PBS. Coverslips were then mounted on slides and imaged on Zeiss fluorescence microscopes.

RNA isolation and real-time PCR

Cells were collected and resuspended with Trizol reagent (Cwbiol #cw0580A). Total RNA was isolated by chloroform extraction and isopropanol precipitation. The pellets were washed with 75% (v/v) ethanol. cDNA was synthesized with a reverse transcription kit (Transgen #AT311). cDNAs of the target gene were quantified by real-time quantitative PCR (qPCR) using SYBR Green PCR Master Mix (Bio-Rad #1725121) on a CFX96TM Real-Time System (Bio-Rad). The transcript level of each gene was normalized to *ACTB*. The following primers were used:

*ACTB*: 5’AGAGCTACGAGCTGCCTGAC and 5’AGCACTGTGTTGGCGTACAG;

*ASNS*: 5’CTGCACGCCCTCTATGACAA and 5’GGAGTCCAAGCCCCCTGATA;

*BIP*: 5’CATCAACGAGCCTACGGCA and 5’ AGACACATCGAAGGTTCCGC;

*CHOP*: 5’TTGCCTTTCTCCTTCGGGAC and 5’TGATTCTTCCTCTTCATTTCCAGG;

*CLPP*: 5’CAGGTGATCGAGTCCGCCAT and 5’GCTAGCTGGGACAGGTTCTG;

*LONP1*: 5’CCGCAACTACCTAGACTGGC and 5’AACGGCAATGAACTCCAGGAT;

*PTPN21*: 5’AAAGTGCTTCTTCCCCTCCC and 5’AATGACGCCAAACATGCAAC;

*YME1L1*: 5’ CCCAGGGACTGGAAAGACAC and 5’ GAGCATTCGCCTTTGCTTCC.

**Amino acid treatment**

For amino acid starvation, cells were incubated in a homemade DMEM base medium devoid of amino acids for 50 min, followed by stimulation with a high-concentration amino acid solution for 30 min. The high-concentration amino acid solution is a mixture of commercial 50× glutamine-free amino acid mixture (Gibco, 11130051) and 100× glutamine (Gibco, 25030081).

**Global protein synthesis assay**

Cells were treated with 50 μg/mL antimycin for 4 h, followed by incubation with 10 μg/mL puromycin for 30 min. Western blot analysis was then conducted using an antibody against puromycin to assess its incorporation into newly synthesized proteins.

**Mitochondrial isolation from** **cultured cells**

HEK293T cells were transfected with pcDNA3.3-based expression vectors and cultured for protein expression. Before collection, the cells were treated with antimycin for 3 h. Cells were then lysed and processed as described above. The Mitochondria Isolation Kit for Cultured Cells (Thermo, 89874) was utilized to isolate mitochondria from HEK293T cells following the manufacturer’s instructions. Both mitochondrial and cytosolic fractions were collected for immunoblotting analysis.

Soft agar colony formation assay

To prepare different concentrations of agarose, a low melting point agarose (Sigma) was used. For the bottom-layer agarose medium, 1% low melting point agarose and pre-warmed 2× culture DMEM medium (Gibco) were mixed in a 1:1 ratio. The mixture was added to a 6-well plate (1.5 mL per well) and solidified at 20°C for 30 min. For the top-layer agarose medium, 0.6% low melting point agarose and cells in a 1× culture medium were mixed. The mixture was poured onto the solidified bottom medium and allowed to solidify at 20°C for 30 min. For MCF7 and SK-BR-3 cells, 8,000 cells were added to each well. The dish was cultured at 37°C with 5% CO_2_ after solidification. 100 μL per well of 1× culture medium was added twice weekly onto the top medium to prevent drying. Incubation was continued for two weeks to ensure adequate colony formation. Colonies were stained with 0.005% crystal violet in 5% methanol and quantified using Image J.

Measurement of oxygen consumption rate (OCR)

Mitochondrial function was evaluated using the Agilent Seahorse XF Cell Mito Stress Test Kit and Seahorse XFe96 FluxPak (Agilent Technologies, 103015-100 and 102601-100). OCR was monitored using the Seahorse XFe96 Analyzer (Agilent Technologies) following the manufacturer's instructions. HEK293T cells (1×10^4^) with the specified genotypes were seeded in a 96-well plate one day prior to the assay, with three replicate culture wells per run. One hour before the assay, cells were washed twice with Seahorse XF DMEM medium (Agilent Technologies, 103575-100) and supplemented with 135 μL of Seahorse XF DMEM medium per well. The medium was further supplemented with 1 mmol/L pyruvate, 2 mmol/L glutamine, and 25 mmol/L glucose (Agilent Technologies, 103578-100, 103579-100, and 103577-100). The cells were then placed in a CO_2_-free BioTek Cytation 1 imaging reader, and bright-field images were captured for each well. The mitostress test was conducted following the standard protocol, using the recommended concentrations of 1 μmol/L oligomycin and 1 μmmol/L rotenone plus antimycin A (Rot/AA). The optimal concentration of carbonyl cyanide-p-trifluoromethoxyphenylhydrazone (FCCP) was determined by titration from 0.125 to 1 μmol/L and found to be 1 μmol/L. To assess cell numbers, protein concentrations were quantified for normalization using the Pierce BCA Protein Assay Kit (Thermo Fisher). The normalization was set to a standardized unit of 1 μg. The resulting cell numbers were imported into the Wave 2.6.1 software, and normalization was applied. Analysis was performed using the Seahorse XF Mito Stress Test Report Generator 4.03.

Labeling of mitochondria

A cell-permeable MitoTracker® probe (Invitrogen™, M7512) was used to label mitochondria. Before the experiment, MitoTracker was dissolved in DMSO to a final concentration of 1 mmol/L. MitoTracker stock solution (1 mmol/L) was diluted to the final working concentration (100 nmol/L) in the growth medium. Cells were grown on coverslips to the desired density. The growth medium was removed from the dish and a prewarmed staining solution containing MitoTracker was added. Cells were then incubated at 37℃ for 30 min. After the staining was complete, the medium was replaced with fresh prewarmed medium and the cells were imaged. Or, cells were fixed with 4% paraformaldehyde and observed with a Zeiss confocal microscope (LSM 710).

**Bioinformatic analysis of *WDR26* expression**

Data on the *WDR26* gene copy numbers in different cancers were compiled from the cBioPortal database. The cBioPortal website serves as a comprehensive platform that integrates data from 126 tumor genome studies, including prominent projects like the Cancer Genome Atlas (TCGA) and the International Cancer Genome Consortium (ICGC). The dataset encompasses 28,000 samples. Additionally, these samples may include valuable information regarding clinical prognosis and other phenotypic characteristics. Upon accessing the website, the target gene was directly entered, and the relevant database was selected to generate a chart showing genetic alterations involving the target gene. The expression levels of *WDR26* in breast cancer and normal tissues were obtained from the GEPIA 2 database.

**Subcutaneous xenograft tumor growth**

Breast cancer cells were trypsinized, washed twice with PBS, and resuspended in PBS. For each injection, 100 µL of the cell suspension was mixed with 100 µL of Matrigel (Corning) and subcutaneously injected into the right flank of a 5-week-old female BALB/c nude mouse (Charles River) using an insulin syringe (BD Biosciences). The number of MCF7 cells was 2 × 10^6^ per injection. Xenografts were measured with a digital caliper every other day since the 6^th^ day after transplantation (tumor volume = width^2^ × length × 0.523). On the final day, the xenografts were dissected, snapped, and weighted. At the request of the Institutional Animal Care and Use Committee (IACUC) at Pony Testing International Group, the tumor size did not exceed 15 mm in its largest diameter. None of the xenograft tumors exceeded this limit. To ensure randomization for experimental analysis, mice were chosen in an unbiased manner. All the animal protocols used in this study were evaluated and approved by the IACUC at Pony Testing International Group.

**Statistical analysis**

Statistical analyses were performed with GraphPad Prism 9.0. Sample treatment, replicates (*n*), quantifications, and statistics are indicated in the figures, figure legends, and methods. Student’s *t*-test and one-way ANOVA were used to calculate the *P* values. Experiments yielding quantitative data for statistical analysis were performed independently at least twice, all with similar results.

Supplementary figures


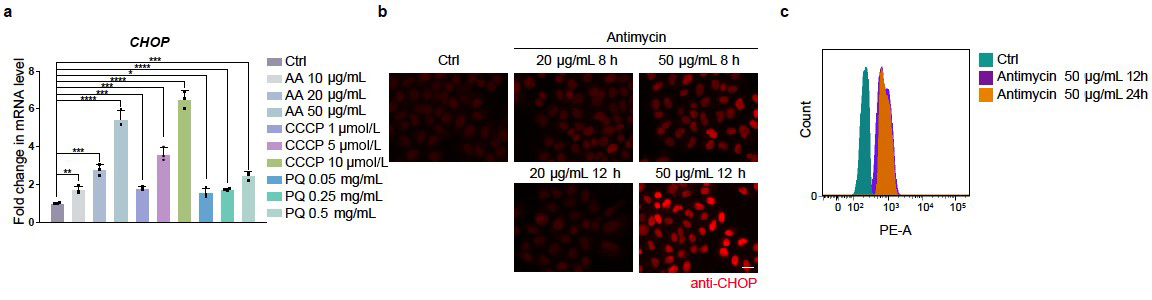
Supplementary Figure S1 Treating cells with mitochondrial inhibitors induces the expression of CHOP. (a) qRT-PCR measures mRNA levels of *CHOP* in cells treated with different concentrations of mitochondrial inhibitors. AA, antimycin; PQ, paraquat. Quantifications are normalized to *ACTIN*. Data are presented as mean ± SEM; *n* = 3 biological replicates. ^*^*P* < 0.05, ^**^*P* < 0.01, ^***^*P* < 0.001, ^****^*P* < 0.0001 by two-sided Student’s *t*-test. (b) Representative fluorescence images of anti-CHOP immunostaining in cells treated with different doses of antimycin. Scale bar, 50 µm. (c) Flow cytometry can separate cells cultured under normal conditions or mitochondrial stress conditions based on anti-CHOP immunostaining.


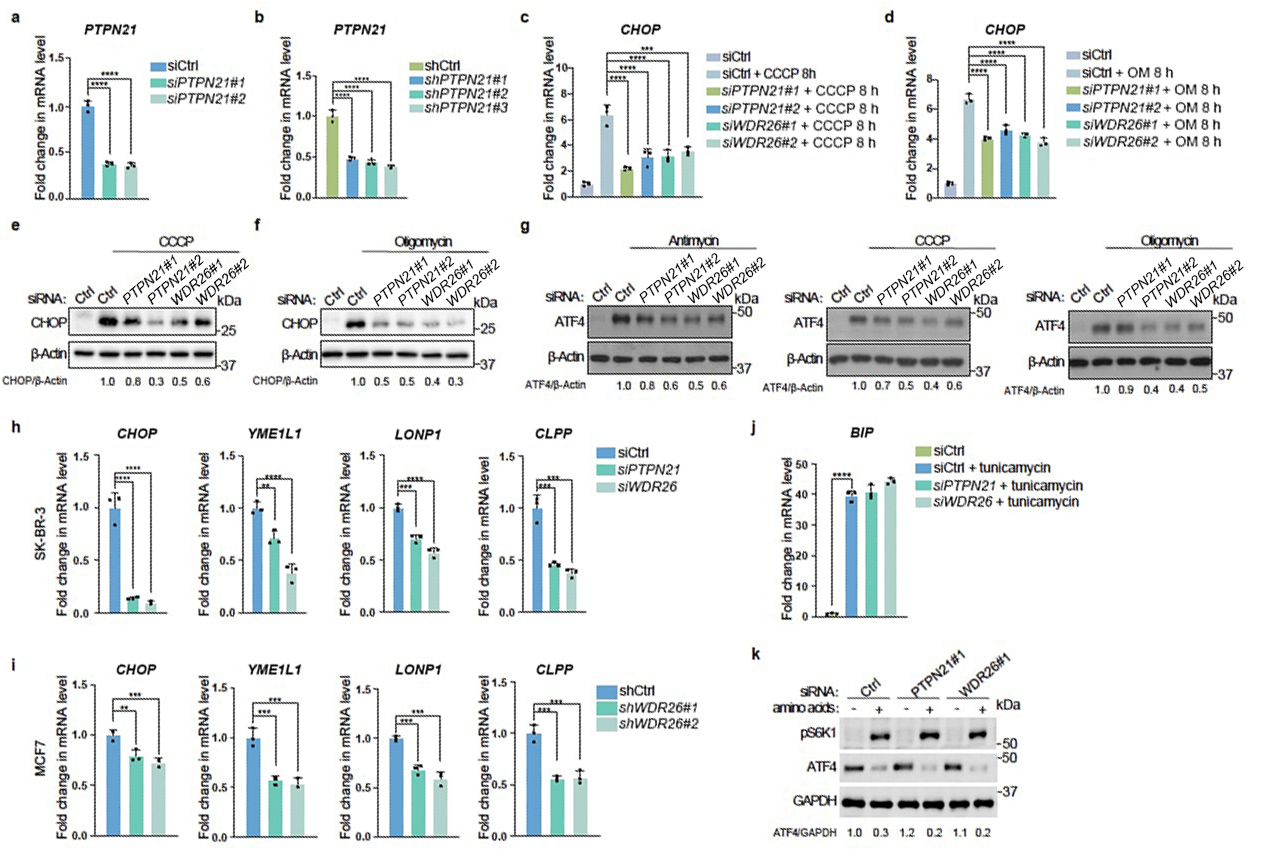


Supplementary Figure S2 Knockdown of *PTPN21* or *WDR26* impairs the induction of mitochondrial stress response genes. (a and b) qRT-PCR measures the endogenous *PTPN21* mRNA level when *PTPN21* is knocked down by siRNA (a) or shRNA (b). (c and d) Knockdown of *PTPN21* or *WDR26* by siRNA suppresses the induction of *CHOP* upon CCCP or oligomycin (OM) treatment. CCCP, 10 μmol/L 8 h; oligomycin, 10 μmol/L 8 h. (e and f) Immunoblotting experiments reveal that knockdown of *PTPN21* or *WDR26* suppresses the induction of CHOP upon CCCP or oligomycin treatment. (g) Knockdown of *PTPN21* or *WDR26* by siRNA suppresses the induction of ATF4 upon CCCP or oligomycin treatment. (h and i) qRT-PCR measures the mRNA levels of mitochondrial stress response genes in SK-BR-3 and MCF7 cells treated with control, *PTPN21,* or *WDR26* RNAi groups. (j) qRT-PCR measures the mRNA levels of *BIP* under the indicated treatments. (k) Knockdown of *PTPN21* or *WDR26* did not inhibit the activation of the ISR under nutrient-deprived conditions. Quantifications are normalized to actin. Data are presented as mean ± SEM. *n* = 3 biological replicates. ^**^*P* < 0.01, ^***^*P* < 0.001, ^****^*P* < 0.0001 by one-way ANOVA test.


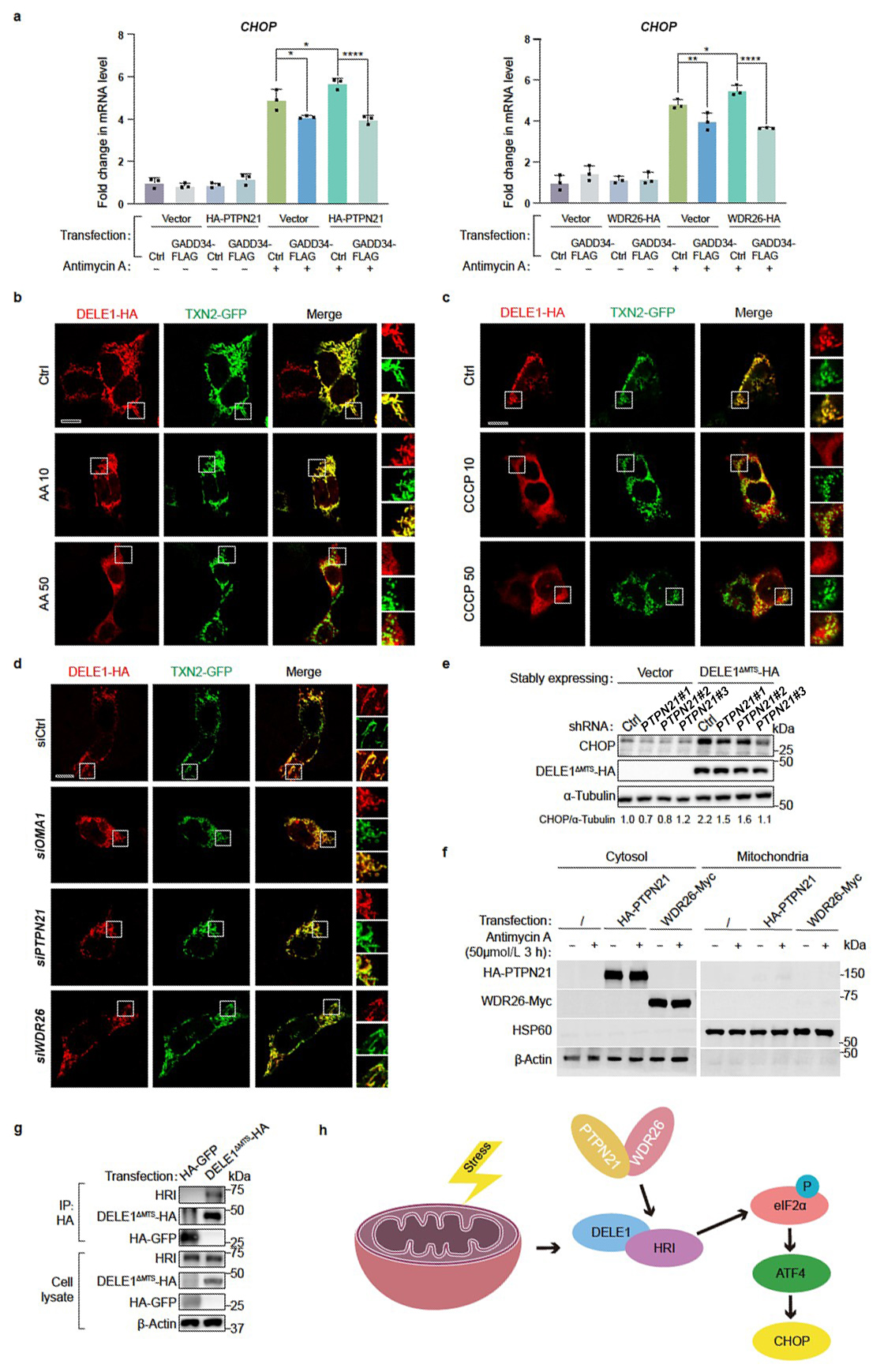


Supplementary Figure S3 PTPN21 and WDR26 promote DELE1-HRI interaction. (a) The elevated induction of CHOP in *PTPN21* or *WDR26*-overexpressing cells treated with antimycin is compromised by *GADD34* overexpression. Quantifications are normalized to actin. Data are presented as mean ± SEM. *n* = 3 biological replicates. ^*^*P* < 0.05, ^**^*P* < 0.01, ^****^*P* < 0.0001 by one-way ANOVA test. (b and c) Representative fluorescence images of DELE1 localization in HeLa cells untreated or treated with different doses of antimycin (b) or CCCP (c). AA 10: antimycin 10 μg/mL; AA 50: antimycin 50 μg/mL. CCCP 10: CCCP 10 μmol/L; CCCP 50: CCCP 50 μmol/L. Scale bar, 20 µm. (d) Representative fluorescence images of DELE1 localization in HeLa cells transfected with control siRNA, or siRNA against *OMA1*, *PTPN21*, or *WDR26*. Scale bar, 20 µm. (e) Knockdown of *PTPN21* suppresses the induction of CHOP caused by overexpression of *DELE1^ΔMTS^*. (f) PTPN21 and WDR26 constantly localize in the cytosol, regardless of the presence or absence of mitochondrial stress inducers. Antimycin, 50 μg/mL 3 h. (g) The cleaved DELE1 interacts with HRI. (h) PTPN21 and WDR26 facilitate the interaction between HRI and DELE1, thereby promoting eIF2α phosphorylation and CHOP expression.


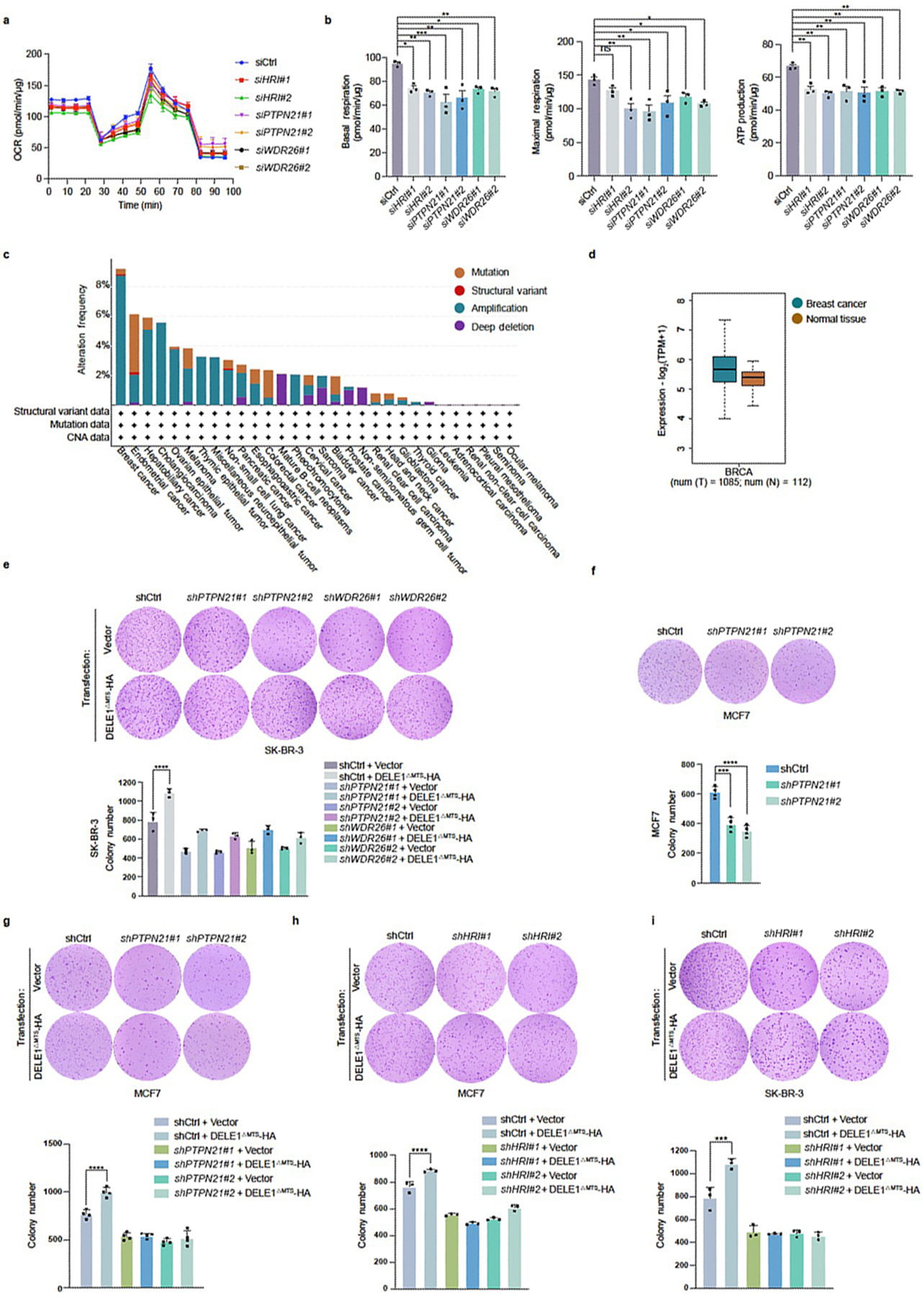


Supplementary Figure S4 PTPN21 and WDR26 control cell fitness. (a) Wild-type or *PTPN21*-, *WDR26*-, or *HRI*-deficient cells were treated with different mitochondrial stressors and the oxygen consumption rate (OCR) was monitored. (b) Basal respiration, maximal respiration, and ATP production were calculated for the indicated cells. *n* = 3 biological replications. Data are presented as mean ± SEM. ^*^*P* < 0.05, ^**^*P* < 0.01, ^***^*P* < 0.001 by one-way ANOVA test. (c) Analysis of the cBioPortal database reveals that breast cancers have the highest number of genomic copies of *WDR26*. (d) Analysis of the GEPIA 2 database reveals that the mRNA level of *WDR26* is higher in breast cancer cells than in normal breast tissue. (e) Deficiency of *PTPN21* or *WDR26* suppresses anchorage-independent growth of SK-BR-3 cells. Colony numbers are quantified. *n* = 3 biological replicates. ^****^*P* < 0.0001 by one-way ANOVA test. (f) Deficiency of *PTPN21* suppresses anchorage-independent growth of MCF7 cells in soft agar. *n* = 4 biological replicates. ^***^*P* < 0.001, ^****^*P* < 0.0001 by one-way ANOVA test. (g) Overexpression of cleaved DELE1 promotes anchorage-independent cell growth in wild-type cells, but not in *PTPN21*-deficient cells. *n* = 4 biological replicates. ^****^*P* < 0.0001 by one-way ANOVA test. (h and i) Deficiency of *HRI* suppresses anchorage-independent growth of MCF7 (h) or SK-BR-3 (i) cells. *n* = 3 biological replicates. ^***^*P* < 0.001, ^****^*P* < 0.001 by one-way ANOVA test.

**Supplementary Table S1** List of the enriched sgRNAs from the “CHOP-low” group.

**Supplementary Table S2** List of the enriched sgRNAs from the “CHOP-high” group.
